# Exploring Personal Support Worker Education, Recruitment, and Retention in Long-Term Care Homes in Ontario, Canada: Protocol for a Mixed Methods Study

**DOI:** 10.2196/79164

**Published:** 2026-04-15

**Authors:** Alison Kernoghan, Danielle Just, Catherine E Tong, Linda Sheiban Taucar, D’Arcy Kirkwood, Benjamin Jay Smith, Living Classroom Team, Angela Butt

**Affiliations:** 1 Research and Knowledge Mobilization, Schlegel-UW Research Institute for Aging, Building 4, 250 Laurelwood Drive, Waterloo, ON, N2J0E2, Canada, 1 519-904-0660; 2 Centre for Health Care Research & Innovation, Conestoga College, Kitchener, ON, Canada; 3 See Acknowledgments

**Keywords:** personal support worker, long-term care, recruitment, retention, education, integrated learning

## Abstract

**Background:**

Long-term care (LTC) homes across Canada are facing a crisis in worker recruitment and retention. In the province of Ontario, personal support workers (PSWs) make up 58% of the LTC workforce and play a key role in addressing staffing shortages. Despite the provincial training and education standards implemented in 2014, PSWs working in LTC consistently report feeling unprepared and undersupported and a lack of confidence, leading to high turnover rates. Integrated learning approaches such as the Living Classroom may ease the transition from education to practice, supporting recruitment and retention of PSWs into LTC.

**Objective:**

This study aims to explore potential relationships between Living Classroom models of PSW training and their recruitment and retention in LTC homes in Ontario, Canada, as compared to traditional, or non–Living Classroom, models and how the experience of the Living Classroom impacts the recruitment and retention of these workers.

**Methods:**

We will use a convergent mixed methods study design in which quantitative survey data and qualitative interview data will be collected concurrently. The quantitative arm of the study will recruit new PSW graduates from both Living Classroom and non–Living Classroom programs, delivering online surveys at 1, 6, and 12 months after program completion to assess demographics, employment status and placement, intent to leave, and the changes in those metrics over time. The qualitative arm of the study will recruit PSW students within 8 weeks of program completion for 60-minute semistructured interviews conducted online via Zoom, over the telephone, or in person. Interview transcripts will be analyzed using thematic clustering and line-by-line emergent coding techniques as well as team-based analysis meetings. In addition to an emergent thematic analysis, we will use a priori codes to capture concepts from the quantitative measures, including recruitment into and intent to leave health care and LTC. Data collection and analysis from both arms will be presented in a side-by-side joint display.

**Results:**

This research is funded by the Ontario Ministry of Long-Term Care from April 2024 to March 2026. Enrollment began on March 3, 2025, and is estimated to be completed by December 31, 2025. As of June 16, 2025, we had enrolled 35 participants for interviews and 132 participants in the surveys. Data collection and analysis are currently underway, and the first results are expected to be submitted for publication in 2026.

**Conclusions:**

The results of this study will inform our understanding of the Living Classroom model for PSW education and its impact on recruitment and retention of PSWs into LTC in Ontario. This evidence will enable educators, LTC administrators, and policymakers to further develop their approaches to PSW training and education as one of many vectors to address the staffing shortages faced by the LTC sector.

## Introduction

### Background

Like many of their international equivalents, long-term care (LTC) homes across Canada are facing concurrent challenges, including a health human resource crisis, rising waitlists, and an aging population increasingly in need of the 24-hour care and congregate living that the sector provides. Worker recruitment and retention is a long-standing issue in LTC, and this was further exacerbated by the COVID-19 pandemic [1]. In the Canadian province of Ontario, where our research is situated, the LTC sector continues to face persistent and sometimes severe staffing shortages [2]. The staff in LTC includes administrators; nurses; allied health professionals; dietary, food, and housekeeping workers; recreational staff; physicians; and others. The largest proportion of LTC staff members are personal support workers (PSWs), who in other jurisdictions are referred to as direct care workers, nurse aides, care aides, and other titles. These support workers are often referred to as “the eyes and ears” of the LTC system [3] given their direct and regular contact with residents.

PSWs are unregulated frontline health care workers who provide direct care to support activities of daily living for individuals across the health care continuum, with most working in LTC, home, and community care settings [4-7]. PSWs are crucial members of the interdisciplinary team in the LTC sector as they are responsible for delivering over 80% of direct care to residents [6]. In Ontario, PSWs make up 58% of the LTC workforce, with most PSWs employed in LTC being female (88.5%) and the average age being 41.2 years [6,7].

Within the Canadian context, escalating pressures to manage the increasingly complex needs of an aging population have significantly evolved the role of PSWs, whose responsibilities have broadened in scope and depth across various clinical settings, including LTC [5,8-11]. Recognizing this expanding scope and the increasing acuity of the residents and patients that PSWs work with [12], in 2014, Ontario introduced education and training standards for the preparation of PSWs [13]. PSW training and certification are provided by community colleges, private career colleges, and continuing education school boards [14]. These programs generally range from 5 to 12 months for full-time students and can extend up to 2 years for part-time students. Courses typically include theory courses, laboratories and practical demonstrations, and a practicum placement in a health care organization (eg, a home care agency or an LTC home). After the pandemic, there has been a notable increase in hybrid PSW training programs in which students complete the bulk of their theory courses online as opposed to classroom work at the school. This broad array of training programs are known as “traditional” PSW education models; however, for the purpose of this study, the term “non–Living Classroom” will be used.

Despite these training and education standards, PSWs working in LTC have consistently reported feelings of being unprepared and undersupported and having a lack of confidence, leading to high turnover rates in LTC [15-22]. In addition to feeling underprepared and lacking confidence, PSWs working in LTC in Ontario also report insufficient staff-to-resident ratios, overworked team members, undertrained colleagues, and high turnover rates that prevent them from providing holistic care [23]. It is in this context that an estimated 40% of PSWs leave the LTC workforce within the first year of graduation and an additional 25% leave after 24 months of work experience [6]. However, Ontario needs more PSWs, not fewer. Researchers project that the Ontario LTC sector will need an additional 11,000 PSWs by 2035 [24], and the government itself projects that the province will need 50,000 more PSWs by 2032 across the continuum of care [25].

Recognizing these challenges, the government of Ontario introduced several initiatives and incentives to attract and retain PSWs in LTC [26]. However, there is a critical need to address PSWs’ experience of feeling unprepared and undersupported and having a lack of confidence in the LTC setting. Innovative education models that better prepare PSWs for the unique demands of the LTC sector may be a solution to the high turnover rates experienced within the first year of employment in LTC [20,27]. The Living Classroom model is one such approach, offering an integrated, hands-on learning experience designed to enhance confidence and competence and longer-term retention in the field.

### The Living Classroom Model for LTC

In September 2009, Conestoga College; the Schlegel-UW Research Institute for Aging; and Schlegel Villages, an LTC home owner and operator, partnered to open their first Living Classroom, where they welcomed a class of 30 PSW students at the Village of Riverside Glen, a retirement community in Guelph, Ontario [28]. They then went on to create a second Living Classroom at the Village at University Gates, which today provides year-round education for PSWs and practical nursing students. Conceptually rooted in the Teaching Nursing Home movement in the United States [29,30], the purpose of this model is to provide PSW education in the context of an LTC home so that students are actively participating in the home while they complete their education. The Living Classroom model takes an interprofessional approach where faculty, students, LTC teams, residents, and families work together within a culture of integrated learning [28]. The goal is to ease the transition from education to practice in LTC and support recruitment and retention. Preliminary research from this model suggests that students in these programs have increased intention to seek employment in LTC [31] and that this approach has supported new PSW graduates in their transition to practice in LTC [32] and increased their gerontological knowledge [33].

### Expansion of the Living Classroom Model Through the Living Classroom Program

In 2023, the Ontario Ministry of Long-Term Care made a significant investment to expand the Living Classroom model in Ontario over 3 years (2023-2026), with the aim to support the training of 1300 new PSWs [34]. The expanded Living Classroom program is delivered on behalf of the province by the Schlegel-UW Research Institute for Aging in collaboration with the Ontario Association of Adult and Continuing Education School Board Administrators. The Living Classroom program had a significant uptake in its first year (2024-2025), doubling the number of Living Classroom PSW education programs in multiple communities across Ontario, including in northern communities and rural and urban settings. Reflecting the diversity of the province, there are francophone and bilingual Living Classrooms (Canada’s 2 official languages are French and English) and Indigenous learning institutes. Reflecting the range of educational offerings across the province, there are Living Classrooms currently associated with public colleges, career colleges, and high school and continuing education school boards that offer PSW training. The Living Classroom models implemented at each site are slightly different and tailored to the students and the community they are supporting.

A program evaluation is being completed to investigate the recruitment and retention of the PSW graduates in LTC through the lens of the students, educational institutions, and LTC homes. The student arm of the evaluation follows students from the beginning of their Living Classroom PSW education to 6 months after their graduation using both surveys and interviews to collect information about their experiences, confidence, and desire to work in LTC. The educational institutions and LTC homes are required to complete a survey at the beginning of their funding period to describe several details, including the delivery model of their Living Classroom, demographic information, educational cohorts, and PSW turnover rates.

### Objectives

The purpose of this study is to explore potential relationships between Living Classroom models of PSW training and PSW graduate recruitment and retention in LTC homes in Ontario, Canada. In addition, we are interested in understanding the experience of the Living Classroom educational model on PSW graduates and how it impacts the recruitment and retention of these workers in the LTC sector.

This study will address the following 2 research questions (RQs):

1. How do PSW graduates from Living Classroom programs compare to PSW graduates from non–Living Classroom programs with respect to their characteristics, recruitment to the LTC sector, and retention within the LTC sector?
2. What are the experiences of PSW graduates from Living Classroom programs, and how does their experience impact their intention to work in LTC and their intent to stay in the LTC setting?

### Terminology and Concepts

This study distinguishes between recruitment, a PSW graduate’s intention to enter the profession, and retention, the longevity of an employee’s term within their job or the LTC sector. To address PSW retention, we consider 2 distinct but related concepts: intent to stay and intent to leave.

These are not opposite measures, and while overlapping variables exist, they are unique concepts [35,36]. Intent to stay is strongly correlated with factors of job satisfaction and is indirectly associated with turnover rates, whereas intent to leave is a stronger and direct predictor of future turnover rates [37].

Intent to leave was selected as the theoretical prospective measure of retention of PSW graduates from Living Classroom programs compared to PSW graduates from non–Living Classroom programs. The focus on intent to leave aligns with the theoretical underpinnings of the Living Classroom model. By providing integrated learning and immersive exposure to the LTC environment during training, the model aims to bridge the gap between education and practice to reduce a graduate’s likelihood of leaving the sector when entering the workforce.

In contrast, other interventions, such as financial incentive programs, are theorized to increase intent to stay by encouraging graduates to commit to working in LTC for a defined period. Together, these concepts clarify how pathways of education and the workplace influence a graduate’s decision to leave or stay within the LTC sector.

## Methods

### Study Design

We will use a convergent mixed methods study design as there is strong congruence between the proposed RQs and the fundamental elements of this study design [38]. The qualitative and quantitative arms of this study will be conducted simultaneously, with both using distinct methods to explore the potential relationships between training PSWs in a Living Classroom and their recruitment and retention in LTC. The data sources from the quantitative and qualitative arms will be analyzed in tandem, allowing for comparison and supporting our interpretation [39]; however, for participants who complete both arms of the study, their datasets will not be linked.

### Setting, Context, and Research Team

Data will be collected in Ontario, Canada, from both PSW students from a Living Classroom program and students who completed their training in a non–Living Classroom program. Data collection for both the qualitative and quantitative arms of the study commenced in March 2025. Qualitative data collection will continue until August 2025, and quantitative data will be collected throughout 2025 and until December 2026, with our team distributing surveys at 1, 6, and 12 months after graduation.

This protocol was developed collaboratively with members of our interdisciplinary research team. This includes early- and late-career research scientists as well as research staff with experience and expertise in mixed methods approaches [40], PSW education and work [41-44], LTC [32,33], and the Living Classroom model itself [33,45]. Our study also draws on the expertise and guidance of a Living Classroom advisory board, which includes representatives from the Ontario Association of Adult and Continuing Education School Board Administrators and LTC homes. This advisory board meets quarterly; they have advised on study design and data collection instruments and, in the future, will support reviewing initial analyses and knowledge dissemination efforts (eg, ensuring readability of plain-language summary reports).

### Participants and Recruitment

#### Quantitative Arm

This study focuses on students in PSW programs in public colleges, school boards, and career colleges in Ontario, who will complete the surveys. Participants will be eligible for the study if they fulfill the following criteria:

- Being currently enrolled in or having recently graduated from a PSW program in Ontario
- Ability to provide informed consent
- Ability to comprehend and respond to questions in English or French
- Access to digital devices to participate in the online survey

We will exclude individuals without access to a computer or cell phone and individuals who did not graduate from a PSW program in Ontario.

We will use a pragmatic, nonprobability sampling approach [46]. We aim to recruit a sample of 250 PSWs, with approximately 125 from a Living Classroom and 125 from a non–Living Classroom program, by emailing administrators to distribute invitations and QR codes to students 1 month before graduation. Interested students will complete an online contact form, after which letters of information, consent forms, and surveys will be digitally distributed. The study information and consent documents will be available in English and French.

We are mindful of the fact that attrition tends to be higher among research participants who have a lower income, are precariously employed, and face other systemic disadvantages [47], and these characteristics reflect some of our anticipated participants.

#### Qualitative Arm

We will use purposive recruitment to obtain a maximum variation sample of 30 to 35 PSW graduates from Living Classroom programs across the province, including from all 5 Ontario Health Regions. The sample ensures representation of geographic spread, delivery format (ie, online, in person, and hybrid), educational partner (ie, private and public colleges and school board programs), and LTC partner (ie, for profit, nonprofit, and municipal). Participants will be drawn from the quantitative arm among those who expressed interest in a follow-up interview. The eligibility criteria for the qualitative component of the study include the following:

- Being currently enrolled in or having recently graduated from a Living Classroom program in Ontario
- Ability to provide informed consent
- Ability to complete an interview in English
- Access to an email account to receive study information

Interviews will be conducted within 8 weeks of graduation. Given resource limitations, interviews will only be available in English. Our sample size is informed by principles of data adequacy, ensuring the capture of rich, diverse perspectives [48].

### Data Collection

#### Quantitative Arm

A survey instrument was developed to collect demographic, predictor, and outcome data using LimeSurvey Cloud (version 6.12.0; LimeSurvey GmbH). Surveys are available in English and French following translation and bilingual review. Each survey takes approximately 10 minutes to complete.

To match responses over time while maintaining anonymity, we use self-generated IDs [49]. Data are collected at 1, 6, and 12 months after graduation to track longitudinal outcomes.

#### Qualitative Arm

Interviews (approximately 60 minutes) will be conducted via videoconferencing, over the telephone, or in person based on participant preference. Interviews will be led by trained interviewers, including 2 PhD-trained qualitative health researchers with prior experience interviewing PSWs and a Master’s-level researcher who is a registered nurse and professor in a college PSW education program. All 3 interviewers are women. A semistructured interview guide will be used (see Textbox 1 for sample questions). The interview will focus on participants’ experiences with the Living Classroom and their professional plans (ie, intent to leave and intent to work in LTC) and job satisfaction. Interviews will be digitally recorded using an external audio recording device; processed through transcription software (MAXQDA; VERBI GmbH); and then cleaned, reviewed, and anonymized by a trained research assistant using established protocols. Audio recordings and raw and cleaned transcripts will be retained on our secure research drive for future analyses.

Textbox 1. **Sample interview guide questions.**

- “Tell me about your experience in your Living Classroom PSW program.”
- “What parts of the program do you feel have been helpful to prepare you for work as a PSW?”
- “Are there aspects of being a PSW that you feel you need more training on or practice with? Please describe.”
- “Do you foresee PSW work as a long-term career for you? Why or why not?”
- “What might lead you to consider leaving work as a PSW?”
- “What do you plan to do when you graduate?” OR “If you have graduated already, are you working currently?”
- “If employed, how did you decide to work in your current job? (e.g., was it where you did your education placement?)”
- “Tell me about your current job satisfaction; what do you like and dislike?”
- “Are you working more than one job? (If yes, probe: the location and sector of other jobs & why they are working multiple jobs)”
- “Was there anything about your education experience that contributed to your plans for work?”

### Data Analysis

#### Quantitative Analysis

Primary outcomes include (1) employment status (binary), (2) employment sector (single selection), and (3) intent to leave (4-point Likert scale). All outcomes will be measured at each time point, and descriptive statistics will be calculated on Living Classroom and non–Living Classroom outcomes to compare employment statuses and sector frequencies, as well as means and SDs of likelihood scale outcomes. Moreover, a McNemar test will be conducted to test for differences in employment statuses between the 2 groups, and a generalized estimating equations model with a logit or probit link function will be performed to test for employment status and temporal interaction effects. A Friedman test will be used to compare between the groups, and a generalized estimating equations model with an ordinal logit link function will be used to compare intent to leave outcomes. All statistical analyses will be performed using Microsoft Excel (version 2511) and R (version 4.5.1; R Foundation for Statistical Computing).

To address missing data due to nonresponses, we will use a combination of listwise or pairwise deletion, multiple imputation (MI), inverse probability weighting, and sensitivity analyses depending on the amount, type, and patterns of missing data as generalized by Mirzaei et al [50]. First, the proportions and patterns of missing data will be analyzed using descriptive statistics, visualization methods, and the Little test to determine the amount, type, and classification of missing data. Second, we will perform listwise deletion under four conditions: (1) if the missing data are demographic outcomes that prevent us from performing paired or subgroup analyses, (2) if there is more than 40% missingness, (3) if it is not possible to address the RQ due to missing data (eg, the participant did not respond to crucial items related to intent to leave or working status), and (4) if missingness prevents a temporal analysis. Third, in similar fashion to the second step, a pairwise deletion will be conducted if a certain item has more than 40% missingness. Fourth, to address nonresponses, MI (eg, MI by chained equations, full information maximum likelihood, and k-nearest neighbor) and inverse probability weighting will be conducted, wherein the specific method will be influenced by the proportions and patterns from step 2. A sensitivity analysis will be conducted to test for robustness given missing not at random data using techniques such as, though not limited to, pattern mixture models and scenario analyses.

#### Qualitative Analysis

We will upload transcripts and field notes or debriefing notes into the MAXQDA software. Following processes that our team has used elsewhere [51], our team-based, thematic qualitative analysis will include 4 steps. The first step is coding and theming the data. Researchers will read through a portion of the dataset (eg, 3-4 transcripts) and create an initial set of codes and definitions. We will then identify themes using a clustering technique [52]. Each cluster will have a proposed name, brief description, illustrative quotations from the data, and a list of codes that support it. In the second step, trained qualitative analysts who were also involved in the interviews will use line-by-line emergent coding techniques as outlined by Saldaña [53] to code the dataset in its entirety. In the third step, extensive memoing and team-based analysis meetings will take place throughout data collection and the theming and interpretation phase. In the fourth step, narrative descriptions will be prepared, with quotes that summarize and illustrate high-level themes. In addition to an emergent thematic analysis, in step 2 (line-by-line coding), we will also include a series of a priori codes and questions to code for to specifically capture concepts that are prominent in the quantitative measures (eg, recruitment to health care and LTC, intent to leave health care and LTC, and satisfaction with the Living Classroom experience). This alignment of core concepts in the quantitative and qualitative datasets will support our mixed methods analysis, which is further detailed below. Throughout this process, we will keep track of how our coding structure and definitions evolve, for example, if an initial code is expanded upon, collapsed, or even deleted. Final reports will use verbatim quotes, in which grammar, natural language, and repeated phrases, among other aspects, will be retained, staying true to participants’ voices [54].

In our analyses, we will report gender when using participant quotes and data. Where appropriate or relevant, we will report findings for the total sample and disaggregate them by gender in accordance with the Sex and Gender Equity in Research guidelines [55]; we will achieve this by specifically coding for gender and the intersection of gender with other statuses (eg, international student status), adopting an intersectional lens [56].

#### Strategies for Rigor

Our strategies for rigor will include retaining a record of methodological and analytical decisions in an audit trail [57], peer reviewing and debriefing [58], clarifying researcher biases [58], and the careful examination of outliers [53]. Our team-based analysis process will benefit from the presence of an interdisciplinary team (PhD-trained qualitative health researchers, a public health professional, and a registered nurse who has taught in PSW programs), each bringing a different lens to the data.

### Concurrent Mixed Methods Analysis

The quantitative outcome measures and qualitative interview questions examine overlapping concepts such as the intent to leave, recruitment, and retention. Following data collection and analysis from both arms, the results will be presented in a side-by-side joint display [59]. Our parallel analyses and presentation of the quantitative and qualitative findings in tandem will facilitate further analysis as a result of confirmation, expansion, or discordance between the 2 forms of data [38].

### Ethical Considerations

This study has been reviewed by and received approval from the Conestoga College Institute of Technology and Advanced Learning Research Ethics Board (586). Informed consent will be gathered as the first item on each questionnaire. Those who do not wish to consent will not continue on to complete the remainder of the questionnaire. Participants in the qualitative arm will have the opportunity to ask any questions and provide verbal or written consent at the start of the interviews after having reviewed the study information letter ahead of the interview. This research focuses on the experiences and perspectives of new PSW graduates, many of whom are international students with precarious status in the country (ie, student visas that will expire when schooling is complete) and uncertain pathways to employment. Many PSWs are racialized women and/or individuals who may have a lower socioeconomic status. As such, we take into consideration the guidance provided by van den Hoonaard and van den Hoonaard [60] on conducting qualitative research with potentially vulnerable populations and the specific guidance by Liamputtong [61] on working with racialized participants and in cross-cultural contexts. For example, our team has protocols and plans in place to respond to a participant who is distressed or expresses an unmet need, and we are mindful of power differentials (eg, not overtly identifying ourselves as PhD-trained scientists), creating a safe space for confidential and authentic conversations (eg, reiterating that any question can be skipped) and using plain language wherever possible. Furthermore, identifying information is not collected through questionnaires or interviews. Where identifying details such as email addresses or names are necessary to contact interview or survey participants, that information will be stored securely and separately from the study data. To support retention in the study, participants receive a CAD $20 (US $15) honorarium via a digital gift card for each of the 3 survey time points completed. Participants in the qualitative arm of the study will receive a CAD $50 (US $36) digital gift card.

### Dissemination

We will present the findings from our research at national, well-attended gerontology conferences (eg, the Canadian Association on Gerontology), as well as relevant industry conferences (eg, Together We Care, This Is Long Term Care, and/or AdvantAge Ontario, annual meetings that together represent nearly all of the LTC operators in Ontario). We will prepare a knowledge brief for decision-makers and a final research report for the program funder. A plain-language summary will be available for study participants. Dissemination will also include peer-reviewed manuscripts in open access journals and accompanying social media posts to highlight new manuscripts as they become publicly available.

## Results

Funding for this research was provided by the Ontario Ministry of Long-Term Care from April 1, 2024, to March 31, 2026; enrollment began on March 3, 2025, and is estimated to be completed by December 31, 2025. As of June 16, 2025, we had enrolled 35 participants for interviews and 132 participants in the surveys. Data collection and analysis are currently underway, and the first results are expected to be submitted for publication in 2026.

## Discussion

### Expected Findings

We anticipate that this study will demonstrate a positive correlation between the Living Classroom model and improved recruitment and retention of PSWs in LTC. Specifically, we anticipate that Living Classroom graduates may report greater confidence and readiness to work in LTC. The immersive nature of the Living Classroom, where students are integrated into the LTC home early and often, is designed to support the socialization of PSW students and may increase their intent to stay.

Existing literature highlights the benefits of the Living Classroom model for student learning [31,62], but there is a gap in longitudinal data that consider employment outcomes and longer-term retention of PSWs. This study builds on previous descriptive work using a convergent mixed methods design to compare graduates from Living Classroom and non–Living Classroom programs. Participants are followed for 12 months after graduation, extending the findings of previous studies to understand the transition of these students to the workforce.

### Strengths and Limitations

A strength of this study is the longitudinal, mixed methods approach, allowing us to quantify recruitment and retention rates while also capturing the nuanced descriptions of the students’ experiences through interviews. The interdisciplinary nature of the research team and the involvement of an advisory board further ensure that the findings are grounded in the practical realities of the LTC sector in Ontario. This work will represent the largest sample to date in any study specific to PSWs in Living Classrooms [31,32,62] and one of the largest samples to date of care aides in the Teaching Nursing Home initiative, where evaluations have focused on resident outcomes, nurses, and schools [63].

We recognize the current limitations of this work, and additional limitations may become apparent as we conduct this research. Due to resource limitations, we are only conducting in-depth interviews in English; as such, the experiences of students in francophone Living Classroom programs may not be represented in the qualitative arm of the study. Second, while we seek to understand students’ intent to stay and retention in the sector, our time-limited study does not allow for longer-term follow-up beyond 12 months. In addition, participant attrition is expected, which may limit our sample sizes and, consequently, the statistical analyses that we are able to perform. Our pragmatic, nonprobability sampling approach means that our statistical analyses are not necessarily generalizable but also reflects known issues with accessing this population [64].

### Future Directions

We are working to secure additional research funding, which would allow us to continue following Living Classroom students for a longer period. Current participants have the option to provide their contact information for potential inclusion in future research. Many of the Living Classrooms that we have recruited from are newly formed and newly funded and, as such, do not yet represent a fully mature Living Classroom program. We anticipate future research and evaluation initiatives to continue assessing the development and longer-term impact of Living Classrooms in Ontario and beyond.

### Conclusions

The results of this study will inform our understanding of the Living Classroom model for PSW education and its impact on recruitment and retention of PSWs into LTC in Ontario. This evidence will enable educators, LTC administrators, and policymakers to further develop their approaches to PSW training and education as one of many vectors to address the staffing shortages faced by the LTC sector.

## Abbreviations

LTC: long-term care
MI: multiple imputation
PSW: personal support worker
RQ: research question
